# Gene therapy clinical trials for childhood blindness and its applications for ocular, auditory and renal ciliopathies

**DOI:** 10.1186/2046-2530-4-S1-O10

**Published:** 2015-07-13

**Authors:** D Chung

**Affiliations:** 1 University of Pennsylvania, Philadelphia, PA, USA

## Purpose

To report the current state of adeno-associated virus mediated retinal gene therapy for inherited retinal degenerations caused by the *RPE65 *gene, and its application to auditory, renal and ocular ciliopathies.

## Methods

In Phase I/II clinical trials, 12 subjects with confirmed mutations in the *RPE65 *gene causing inherited retinal degeneration were given a unilateral sub-retinal injection of AAV.*RPE65 *into the worse seeing-eye. Objective and subjective visual testing were performed at baseline and following intervention. Subsequently, 11/12 subjects received injections in the contralateral eye, in a Phase II follow on study. These results have initiated the first Phase III clinical trial for retinal disease. The methods and technology used in this study have enabled gene transfer to cells involved with ciliopathies. Unilateral subretinal, intra-otocyst, and retrograde ureteral injections of AAV vectors were carried out in cohorts of mice. Intra-otocyst injections were carried out at embryonic day 12; ocular and renal injections were performed in adult mice. The transgene cassette consisted of a CMV-promoted EGFP and/or a luciferase transgene. Transgene expression was evaluated qualitatively and quantitatively over time. Cellular specificity of expression was evaluated histologically. Delivery of the vector was well tolerated locally and systemically and the animals showed no alteration in behavior or general well-being.

## Results

The results from the phase I/II clinical trials showed improvements in one or more visual function endpoints for all subjects. There was no evidence of inflammatory response as a result of surgery or exposure to the vector. In respect to the animal studies for ciliopathies, the cellular specificity and efficiency of transduction differed for each vector/delivery approach. After subretinal injection, vectors delivered high levels of transgene product to ciliated photoreceptor cells. In the otocyst, vectors transduced targeted cochlear hair cells efficiently. Following a trans-ureteral approach, transgene expression in the kidney was visible in the tubule cells of the collecting ducts.

## Conclusions

Initial phases for retinal gene therapy have shown safety and efficacy, which have given rise to the first Phase III clinical trial for retinal degenerative disease, which is ongoing. AAV vectors allow efficient transduction of specific subsets of cells with primary cilia. AAV vectors can efficiently transduce photoreceptors, cochlear hair cells, and renal tubular epithelial cells, and could be used to develop treatments for retinal and cochlear degeneration and inherited kidney disease, respectively. These vectors could also be employed to treat syndromes such as Usher Syndrome, Bardet-Biedl Syndrome and polycystic kidney disease.

